# Genome Sequence and Adaptation Analysis of the Human and Rice Pathogenic Strain *Burkholderia glumae* AU6208

**DOI:** 10.3390/pathogens10020087

**Published:** 2021-01-20

**Authors:** Zhouqi Cui, Sai Wang, Kaleem Ullah Kakar, Guanglin Xie, Bin Li, Gongyou Chen, Bo Zhu

**Affiliations:** 1 Key Laboratory of Urban Agriculture by Ministry of Agriculture of China, School of Agriculture and Biology, Shanghai Jiao Tong University, Shanghai 200240, China; zhouqicui@gmail.com (Z.C.); wangsai@sjtu.edu.cn (S.W.); gyouchen@sjtu.edu.cn (G.C.); 2 Department of Plant Pathology & Ecology, the Connecticut Agricultural Experiment Station, New Haven, CT 06511, USA; kakar1002@126.com; 3 State Key Laboratory of Rice Biology, Institute of Biotechnology, Zhejiang University, Hangzhou 310027, China; glxie@zju.edu.cn (G.X.); libin0571@zju.edu.cn (B.L.)

**Keywords:** *Burkholderia glumae*, bacterial panicle blight, chronic granulomatous, comparative analysis, horizontal gene transfer

## Abstract

*Burkholderia glumae* causes rice (*Oryza sativa*) bacterial panicle blight, which is an increasingly economically important disease worldwide. As the first *B. glumae* strain isolated from patients with chronic infections, AU6208 has been reported as an opportunistic clinic pathogen. However, our understanding of the molecular mechanism underlying human pathogenesis by *B. glumae* remains rudimentary. In this study, we report the complete genome sequence of the human-isolated *B. glumae* strain AU6208 and compare this to the genome of the rice-pathogenic *B. glumae* type strain LMG 2196^T^. Analysis of the average nucleotide identity demonstrated 99.4% similarity between the human- and plant-pathogenic strains. However, the phenotypic results from this study suggest a history of niche adaptation and divergence. In particular, we found 44 genes were predicted to be horizontally transferred into AU6208, and most of these genes were upregulated in conditions that mimic clinical conditions. In these, the gene pair *sbn*AB encodes key enzymes in antibiotic biosynthesis. These results suggest that horizontal gene transfer in AU6208 may be responsible for selective advantages in its pathogenicity in humans. Our analysis of the AU6208 genome and comparison with that of LMG 2196^T^ reveal the evolutionary signatures of *B. glumae* in the process of switching niches from plants to humans.

## 1. Introduction

*Burkholderia glumae* is a Gram-negative plant pathogen that causes bacterial panicle blight (BPB) in rice (*Oryza sativa*) and wilting in a wide range of economically important vegetables such as pepper (*Capsicum* sp.), eggplant (*Solanum melongena*), and tomato (*Solanum lycopersicum*) [1,2]. To date, BPB has been reported in more than 21 countries throughout South and Central America, Africa, and Asia [3,4,5,6,7,8], causing costly damage (up to 75% yield reduction) to the rice industry [1].

To date, many rice-pathogenic *B. glumae* strains with different virulence levels have been identified in different countries [9]. Genome comparisons revealed weak variation in virulence-related genes between the strain BGR1 and the high-virulence strain 336gr-1 [1,10]. Later, it was proposed that the varied strains and broad host ranges of *B. glumae* may have been generated by the rapid rearrangement, inversion, or deletion of virulence-related genes [11]. Although comparative omics studies of *B. glumae* will provide evolutionary clues for exploring the unknown host–bacteria interactions, as well as the overall infection process, we lack comprehensive studies on the human-pathogenic *B. glumae* strain AU6208, which was isolated from patients with chronic granulomatous disease [12].

To understand the differences between *B. glumae* strains from different host niches, we generated and analyzed the complete genome sequence of the human-pathogenic strain AU6208. Interestingly, AU6208 was reported to be more virulent on rice than the rice-pathogenic strain BGR1 [13]. This result suggested that this human-pathogenic strain may have strong niche adaptation. Therefore, we investigated several interesting features pertaining to its virulence, evolution, and response to selective pressure by comparing AU6208 to the rice-pathogenic strain LMG 2196^T^. Our results can help to determine the evolutionary changes that enable *B. glumae* adaptation for growth on different hosts.

## 2. Results and Discussion

### 2.1. The Genetic Relationship between AU6208 and LMG 2196^T^

**Figure 1:**
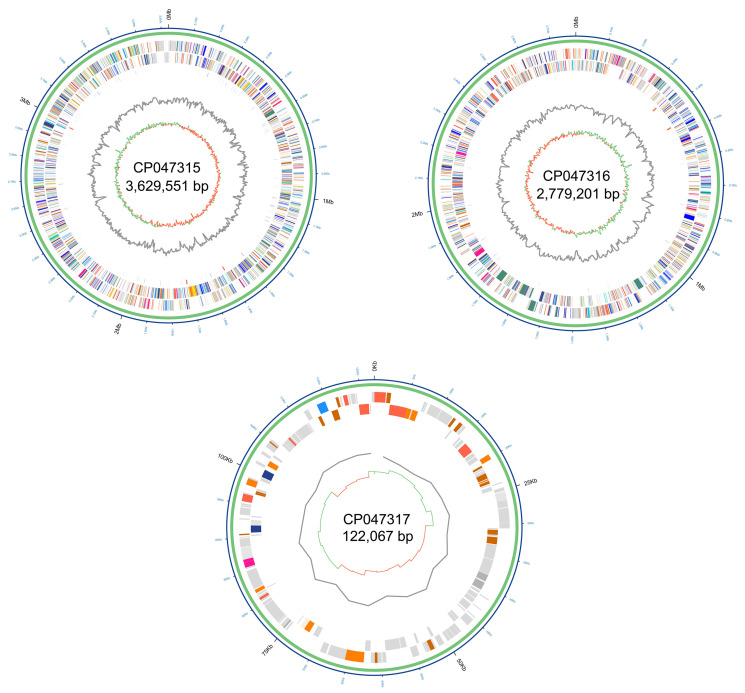
Circular representation of the complete genome of *Burkholderia glumae* strain AU6208. Rings from outside to the center: *B. gluma*e AU6208 chromosome or plasmid, genes mapped against Clusters of Orthologous Groups (COG) database, RNA sequences, GC skew, and GC content.

**Figure 2:**
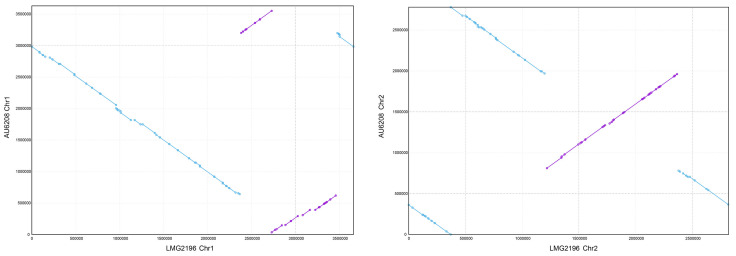
Genetic synteny visualized by MUMmer alignments. A comparison of the chromosomes of AU6208 and LMG 2196^T^. The conserved gene synteny and some rearrangement between the chromosomes are observed. The purple dots represent the synteny genes between the two strains, while the blue dots represent the rearrangement genes. The lines connected by dots represent the high synteny regions (purple lines) or rearrangement (reverse arrangement) regions (blue lines).

To better understand the unique features of the human-pathogenic strain AU6208, we first sequenced its entire genome and analyzed general features of the full-length sequenced genome and assembly information (Table 1 and Figure 1). Based on Pacbio-Illumina hybrid sequencing, a total of 78,783 (14 Kb average read length) and 4,779,545 (2X150 bp pair-end) high-quality filtered reads were obtained from Pacbio and Illumina, respectively. The size of the *B. glumae* AU6208 whole genome is 6.06 Mbp and the genome contains 5008 predicted coding sequences (CDSs), which are located on two chromosome DNA and one plasmid. Strikingly, the average nucleotide identity (ANI) between AU6208 and LMG 2196^T^ was 99.45%, suggesting an extremely high genetic similarity. The synteny analysis between the two strains also suggested the high synteny with some large-scale rearrangement (Figure 2). In total, 257 genes were found to be unique in AU6208 compared to LMG 2196^T^ (Table S1). Most of these genes are annotated as hypothetical protein. Notably, the whole genome of *B. glumae* AU6208 contained 147 and 4 genes related to virulence and antibiotic resistance, respectively (Tables S2 and S3). Interestingly, the number of virulence- and antibiotic-resistance-related genes in LMG 2196^T^ is virtually identical to that of AU6208, except that AU6208 contains two additional virulence-related genes encoding pmlR/bspR1 and BCAL3235 (Tables S2 and S3), which play important roles in capsule polysaccharide biosynthesis in *Burkholderia cecocepacia* [14].

### 2.2. AU6208 Showed Increased Biofilm Formation, EPS Secretion, and Virulence

**Figure 3:**
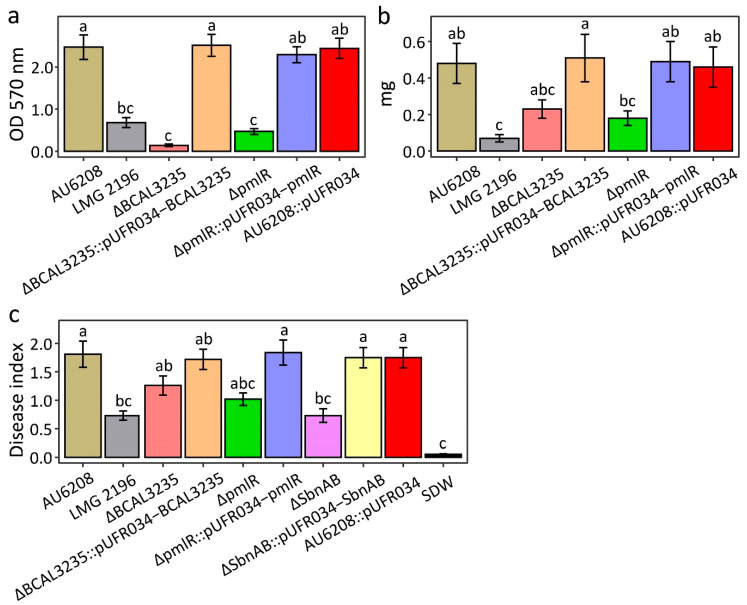
Biofilm formation, exopolysaccharide (EPS) production, and pathogenicity of *B. glumae* strains AU6208, LMG 2196^T^, AU6208^ΔBCAL3235^, AU6208^ΔSbnAB^, AU6208^ΔpmlR^, AU6208- pUFR034 empty vector control and their complementary strains. (**a**) Biofilm formation after a 48 h incubation; (**b**) EPS quantification after a 24 h incubation; (**c**) disease index in rice. All these experiments were repeated three times with four replicates. SDW: distilled water. Different letters denote statistical significance (*p* < 0.05, identified by analysis of variance comparisons of means, employing a posthoc Tukey-Cramer test for multiple comparisons).

As mentioned above, *pmlR/bspR1* and BCAL3235 may give AU6208 some advantages in biofilm formation and exopolysaccharide (EPS) secretion compared to LMG 2196^T^. To confirm this hypothesis, we measured biofilm formation and EPS secretion in AU6208^WT^, AU6208^ΔpmlR^ and the AU6208^ΔBCAL3235^ strains. The morphological appearance and thickness of the biofilm showed obvious differences after 48 h of adhesion at 30 °C without agitation (Figure 3). In particular, the AU6208 strain formed a thicker biofilm compared to the AU6208^ΔpmlR^ and AU6208^ΔBCAL3235^ strains. When quantified, WT (wild type) yielded readings at least four times as high as AU6208^ΔpmlR^ and AU6208^ΔBCAL3235^ (Figure 3). The weight of EPS from AU6208^WT^ was at least twice as heavy as that from AU6208^ΔpmlR^ and AU6208^ΔBCAL3235^ (Figure 3). The quantitative data confirmed that AU6208 exhibited a significantly (*p* < 0.001) higher biofilm-forming capability and EPS secretion compared to AU6208^ΔpmlR^ and AU6208^ΔBCAL3235^. The above results strongly suggest that the *pmlR* and *BCAL3235* gene in AU6208 contributes to increased biofilm formation and EPS secretion. In the virulence test in rice, we found that AU6208^ΔpmlR^ and AU6208^ΔBCAL3235^ showed slightly decreased virulence compared to the WT (Figure 3). The complement strain showed a similar result compared to the WT (Figure 3).

Interestingly, *pmlR/bspR1* is a LuxR family transcriptional regulator, which may be involved in quorum sensing (QS). Devescivi et al. (2007) characterized the important role of QS in the strong rice virulence of *B. glumae* AU6208. They found that, compared with *B. glumae* LMG 2196^T^, the active TofR (a LuxR family regulator) present in AU6208 is the reason to cause strong disease symptom in rice [13]. In this study, since we achieved the whole sequences of both strains, the result of comparative genomic analysis presented that the *pmlR/bspR1* is unique for AU6208 but not in LMG 2196^T^. Since the *pmlR/bspR1* has been demonstrated as the key regulator responsible for the expression of LuxR and virulence of *Burkholderia pseudomallei* [15], it is reasonable to suggest that *pmlR/bspR1* regulated LuxR system plays a key role in virulence of strain AU6208, which in terms that the weak virulence of LMG 2196 ^T^ may be because of the incomplete LuxR system (QS). Moreover, since the QS is responsible for the biofilm formation, the data in this study also suggested that the presence of active *pmlR/bspR1* in *B. glumae* is critical for the formation of biofilm matrix (Figure 3). This is in agreement with reports for many human pathogens in *Burkholderia* family. For instance, it is well demonstrated that *pmlR* is critical for full virulence of *Burkholderia pseudomallei*, the causative agent of melioidosis [15]. In this respect, the presence of the QS system appears to be an indicator of the virulence capacity of *Burkholderia* family, at least of *B. glumae.*

Besides *pmlR/bspR1*, BCAL3235 is another unique gene present in AU6208 compared to LMG 2196T. *BCAL3235* encoded UDP-galactopyranose mutase (UGM) is a unique flavin-dependent enzyme, which converts UDP-galactopyranose (UDP-Galp) to UDP-galactofuranose (UDP-Galf), the major component of cell surface glycoproteins and glycolipids [16]. UGM presents in a variety of pathogenic fungi and bacteria, such as *Aspergillus, E. coli, Klebsiella*, and *Mycobacteria* [17]. The UGM has been identified in *Burkholderia cepacian* complex [18], which is a common causal agent of human chronic granulomatous disease. Thus, the finding of *BCAL3235* in *B. glumae* strain AU6208 but not rice pathogen LMG 2196^T^ strongly suggested that the gene may be achieved from bacteria survived in similar niche for adaption.

### 2.3. Horizontal Gene Transfer May Contribute to Niche Adaptation in AU6208

To further understand the niche adaptation in *B. glumae* AU6208, we compared the whole-genome expressed proteins of AU6208 under the human mimic [19] and *in planta* mimic [20] conditions using 2D SDS-PAGE, and the results shown are a representative expression from duplicate experiments (Figure S1). Most of the genes were expressed in both conditions. Notably, 30 proteins were expressed only in one condition (Figure S1). These differentially expressed proteins were digested and further examined by LC–MS/MS and the transcriptomic levels of the corresponding genes were determined by using qRT-PCR. Twenty-two genes showed a dramatic increase in expression under human mimic condition compared to planta mimic condition (Table 2). Of which, two virulence genes (*sbnA* and *sbnB*) were uniquely presented in AU6208. These data suggest that these genes may be responsible for bacterial survival and virulence in human host. To investigate whether these genes are achieved from microorganisms for niche adaption, we conducted a comprehensive horizontal gene transfer (HGT) analysis (Method 3.6) and total 44 genes were predicted to be horizontally transferred to AU6208 from other organisms (Table S4). Furthermore, 12 of the 22 (marked yellow at Table 2) significantly expressed genes (54.5%) belonged to the horizontally transferred genes. Although the transmission of *B. glumae* from rice condition to human condition is still unclear, these data indicated that horizontal gene transfer plays some role in the niche adaption of *B. glumae* strain AU6208.

**Figure 4:**
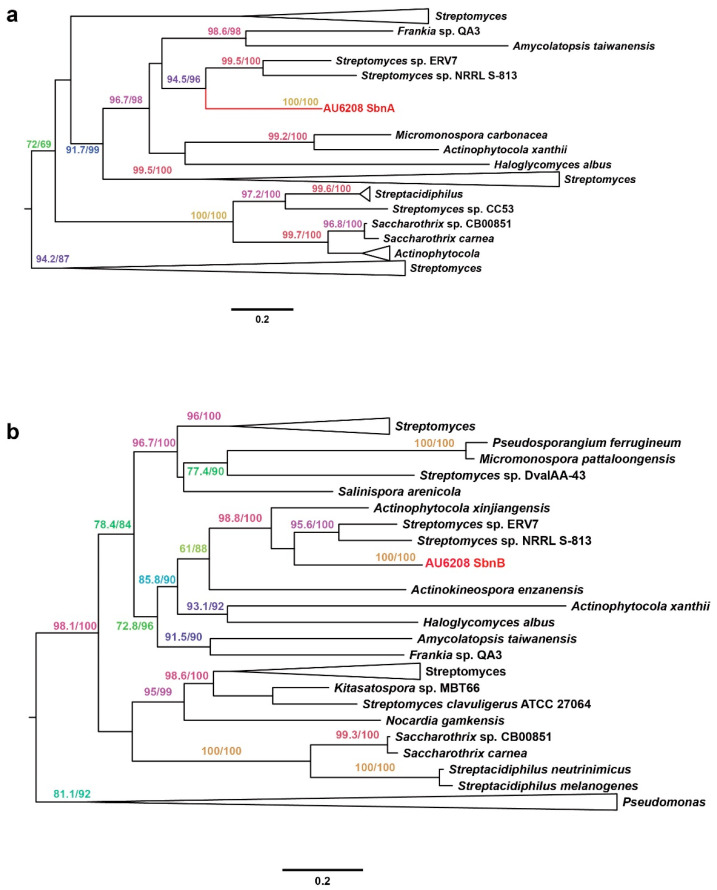
Phylogeny of (**a**) SbnA and (**b**) SbnB. The phylogenetic tree was calculated using the maximum likelihood program PhyML and Bayesian program MrBayes (detailed parameters are described in the main text). Only the values in ML ≥70% and Bayesian posterior probability ≥65% are shown. For key nodes, the actual support values are shown in the order ML bootstraps/Bayesian posterior probability. Bar, 0.1 substitution per site.

Interestingly, we found that a gene pair, *sbnAB*, which is involved in staphyloferrin B biosynthesis, is among the 44 horizontally transferred genes [21]. Phylogenetic analysis of both genes strongly suggests their *Streptomyces* origin with strong bootstrap support (Figure 4). It has been reported that staphyloferrin B is a hydrophilic siderophore, which plays an important role in pathogenicity in both human and plant pathogens [22,23]. Pathogenicity results confirmed that the AU6208^ΔSbnAB^ mutant, which lacks SbnAB, induced no obvious symptoms on the rice plants (Figure 3). The complement strain showed a similar result compared to the WT (Figure 3). This result may be partially explained by the requirement of siderophore biosynthesis in bacteria for plant colonization [24].

Overall in this study, we reported the complete genome of the human-pathogenic *B. glumae* strain AU6208 and compared it with that of the plant-pathogenic strain LMG 2196^T^. This study is the first to report the whole genome of a human-pathogenic *B. glumae* strain, AU6208. Our comparative genomic analysis revealed increased diversity in this pathogen induced by different niche survival needs. We explored the differences in molecular mechanisms of virulence and the difference in the interaction of the human-pathogenic *B. glumae* strain AU6208 and rice-pathogenic *B. glumae* strains with rice. Our results provide insight into the different ecological niches of *B. glumae* strains resulting from differences in virulence and environmental adaptation, as well as their unique interactions with the host during pathogenesis.

## 3. Materials and Methods

### 3.1. Bacterial Strains, Plasmids, and Growth Conditions

*B. glumae* strains and plasmids used in this study are listed in Table 3. All strains were stored at −80 °C in 20% glycerol and grown in nutrient broth (NB) medium (1% peptone, 0.3% yeast extract, and 0.5% NaCl, pH 7.5) at 30 °C. Luria-Bertani (LB) agar without sucrose and LB agar containing 10% (*w/v*) sucrose were used for deletion mutagenesis. Antibiotics were added at the following concentrations when required: 50 µg/mL kanamycin (Km), 100 µg/mL ampicillin (Amp) and 10 ug/mL neomycin (Nm).

### 3.2. DNA Extraction, Genome Sequencing, and Genomic Analysis

Bacterial genomic DNA was extracted using TIANamp Bacteria DNA kits (Tiangen Biotech, Beijing, China). Whole-genome DNA sequencing of *B. glumae* AU6208 was conducted using the Pacific Biosciences (PacBio) RSII Single Molecule Real Time (SMRT) system and Illumina with the NEBNext Ultra DNA Library Prep Kit (New England Biolabs, MA, USA). The combination of PacBio and Illumina sequence shotgun data was assembled with the SMRT analysis packages (v2.1) using hierarchical genome assembly [25]. Low-quality sequences were filtered by Sickle (v1.33) with Q30 (https://github.com/najoshi/sickle). Protein-coding regions, tRNAs, and rRNAs were predicted using rapid annotation subsystem technology [26]. Hits were then analyzed for signal peptide characteristics using SignalP [27]. Transmembrane domains and functional protein domains were analyzed using tied-mixture hidden Markov modeling and InterProScan, respectively [28,29]. The secondary metabolite clusters, antibiotic resistance clusters and toxins, and virulence factors were predicted by comparing with DoBISCUIT, CARD, and VFDB databases, respectively [30,31,32]. The category of functional proteins was further annotated by Clusters of Orthologous Groups (COG) and Gene Ontology databases [33]. Genome comparison between AU6208 and LMG 2196^T^ was conducted using average nucleotide identity (ANI) and alignment fraction (AF), which were calculated by an ANIcalculator [34]. Synteny of genome structure between AU6208 and LMG 2196^T^ was performed by Mummer 3.0 with default parameters [35].

### 3.3. Construction of Deletion Mutants and Complementation

To investigate the role of genes of interest in AU6208, we constructed deletion mutants in AU6208 using homologous recombination. Briefly, two fragments flanking the left and right borders of corresponding genes were amplified from the wild-type genomic DNA with the primer pairs listed in Table S4. The amplified fragments were cloned into pGEM-T (Promega), confirmed by sequence analysis, and then digested and subcloned into vector pKMS1 [36] at *BamH*I and *Pst*I (or *Sal*I) sites. The resulting recombinant plasmids were introduced into AU6208 by electroporation, and transformants were plated on NA plates supplemented with kanamycin. Colonies resulting from a single homologous crossover (integration of the deletion construct at either the left or right border of the target gene) were then transferred to NBN broth, grown for 12 h at 28 °C, and then plated on NAS plates for sucrose-positive deletion mutant selection. Sucrose-resistant colonies were visible within 3 to 4 days and then transferred to NA plates and NA plus kanamycin plates. Kanamycin-sensitive colonies could be mutants containing a second homologous crossover, which was further confirmed by PCR amplification.

In order to complement the deletion mutants, the full-length of corresponding genes including native promotors were amplified using primer pairs listed in Table S5. After confirmation by sequence analysis, the amplified DNA fragments were cloned into pUFR034 [37] at the *BamH*I and *Sal*I sites to create the recombinant plasmids, the recombinant plasmids were transferred into corresponding mutants by electroporation, and transformants were screened on NA plates with neomycin. Colonies were visible within 3 to 4 days growing at 28 °C. A putative transformant was shown to contain plasmid by PCR analysis.

### 3.4. Biofilm Formation and Exopolysaccharide (EPS) Secretion Assay

Biofilm formation was determined using a previously described method [38]. In brief, bacterial cells grown overnight were inoculated 1:100 in fresh NB media in a 96-well microplate (Corning-Costar Corp., Corning, NY, USA). After static culture at 30 °C for 48 h, cells were stained with 1% crystal violet (CV) for 15 min. The planktonic cells were removed by several rinses with H_2_O. The CV-stained bound cells were air-dried for 1 h and dissolved in 90% ethanol, and the optical density at 590 nm (OD_590_) of the solution was measured to quantify biofilm formation.

The extraction and quantitation of bacterial EPS were performed as described previously [39]. Briefly, overnight bacterial cultures were inoculated 1:100 in 100 mL fresh NB media to OD_600_ = 1.0. Cultures were centrifuged at 4 °C, and 100 µl of the supernatant was collected and filtered through 0.22-µm Millipore filters to remove all bacterial cells. A mixture of one volume of supernatant with four volumes of Sevage’s solution (4:1 chloroform:butanol) was incubated with shaking at 180 rpm for 30 min. Subsequently, the supernatants were collected by centrifuging and then mixed with three volumes of 95% ethanol and incubated at 4 °C for 24 h without shaking. The pellet was harvested, dried, and weighed after incubation. The experiments consisted of three biological replicates and were repeated at least three times.

### 3.5. Pathogenicity Assay

In a greenhouse, rice plants (*Oryza sativa* cv. kitaake) were inoculated at the flowering stage with approximately 1 × 10^9^ CFU/mL of *B. glumae*. Disease symptoms in the rice were evaluated on day 10 after inoculation. The disease index was determined as described previously [40]. In detail, the disease score was defined as 0 = healthy panicle, 1 = panicle 0 to 20% discolored, 2 = panicle 20 to 40% discolored, 3 = panicle 40 to 60% discolored, 4 = panicle 60 to 80% discolored, and 5 = panicle 80 to 100% discolored (disease index = $\sum^{\;}$(number of samples per score X score)/the total number of panicles). Pathogenicity assays were repeated three times with four replicates.

### 3.6. Horizontal Gene Transfer (HGT) Analysis

Screening for HGT was first performed by HGTfinder with default parameters [41]. Confirmation of HGT was performed by a combination of BLAST screening and large-scale phylogenetic trees. BLAST screening was performed based on our previous study [42]. In brief, candidate sequences in *B. glumae* were compared against sequences in the NCBI (National Center for Biotechnology Information) reference genome using BLASTp [43] followed by extracting the sequences with highest similarity for further study. We considered two genes orthologs when the E-value <10^−^^10^ and when the alignment similarity was higher than 30% with more than 85% coverage [44]. These sequences were aligned using MAFFT [45], and the conserved region of each alignment was trimmed using TrimAI [46] with stringent settings. Maximum likelihood and Bayesian phylogenies were generated based on our previous study [42].

### 3.7. Preparation of Bacterial Samples Isolated from Different Niches for 2-Dimensional Electrophoresis

For the human mimic condition, overnight bacterial cultures of *B. glumae* AU6208 and LMG2196 from one single colony in NB broth were trans-cultured as 1:100 in 100 mL fresh SCFM2 (Human mimic medium) [19] and incubated with shaking at 200 rpm at 30 °C until the OD_600_ reached 0.8. Cells were then collected by centrifugation at 5000× *g* for 10 min, washed with 0.5 × phosphate-buffered saline (PBS) three times, and used for protein extraction. For the *in planta* mimic condition, we collected the bacterial samples using the previous method [20]. In brief, the rice leaves were collected and homogenized by liquid nitrogen with a mortar and pestle. The resultant was aliquoted to 1 g in tubes and stored at −80 °C before using. The 1 g of freezing rice leaf resultant was thaw on ice and added into 40 mL of nutrient broth to ready as the planta mimic medium. Single colony of *B. glumae* strains was inoculated into the medium and grew to the mid-exponential phase (OD_600_ = 0.5). The bacterial pellets were harvested from 1.5 mL bacterial suspension by centrifugation at 5000× *g* for 20 min, washed with 0.5 × PBS three times, and used for protein extraction. Extraction of total proteins and 2-dimensional electrophoresis were performed as previously described [47]. The resulting protein profiles were analyzed with ImageMaster 2D platinum, version 5.0 (Amersham Biosciences, Uppsala, Sweden).

### 3.8. Protein in-Gel Digestion and Liquid Chromatography–Tandem Mass Spectrometry (LC–MS/MS) Analysis

The tryptic in-gel digestion protocol, optimized for a polyacrylamide gel of 1.5 mm thickness, was performed based on a previous method [48]. Briefly, protein spots were excised from the gel and put into the 96-well polypropylene ZipPlate Micro-SPE plate (Thermo). Then, the gel piece was rinsed for 40 min with 100 μL of 25 mM ammonium bicarbonate/acetonitrile (95:5, *v/v*) followed by two rinses with 100 μL of 25 mM ammonium bicarbonate/acetonitrile (50:50, *v/v*). The gel piece was then dehydrated for 12 min with 200 μL of acetonitrile. Digestion was performed with 15 μL of sequencing grade porcine trypsin (10 ng μL^−1^) at 37 °C for 3 h.

The peptides released from trypsin digestion for LC–MS/MS analysis were prepared in two biological replicates. LC was performed using a Dionex Ultimate 3000 nano-LC system following the method in our previous paper [49]. The MASCOT LC-MS/MS ion search algorithm (Matrix Sciences) was applied to evaluate the LC/MS spectra, and the sequence similarity of resulting peptides to the *B. glumae* AU6208 strain was compared to other *Burkholderia* species accessible on NCBI. To further identify the proteins, the cross-correlation (X corr) scores of singly, doubly, and triply charged peptides were fixed greater than 1.8, 2.5, and 3.5, respectively [49]. Peptide sequences with the highest X corr values were then identified.

### 3.9. Quantitative Real-Time PCR

Total RNAs were extracted from exponentially growing cells, using a RNeasy Mini spin columns Kit (Qiagen) and was treated with a unit of RNase-free DNase I (Qiagen), and cDNA synthesis was performed with a Moloney murine leukemia virus reverse transcriptase first-strand cDNA synthesis kit (QIAGEN). The cDNA was then used directly as the template for qRT-PCR using a SYBER Green master mix (Protech Technology Enterprise Co., Ltd.) on an ABI Prism 7000 sequence detection system (Applied Biosystems). Primers for quantitative real-time PCR (qRT-PCR) of the selected genes were designed by using Primer 3 [50] and *gyrB* was used as an internal control. Ct values were shown on Table S5.

### 3.10. Statistical Analysis

Statistically significant differences in the disease index, biofilm formation and EPS production were identified with a one-way ANOVA and Tukey–Kramer post-hoc test in the agricolae package in R.

### 3.11. Availability of Data and Materials

All data supporting the conclusions of this article are included in this article and its additional files. The complete genome sequences have been deposited at DDBJ/EMBL/GenBank and the bioproject ID is PRJNA595825.

## Figures and Tables

**Table 1:** Genome statistics on *Burkholderia glumae* AU6208.

Feature	Chromosome1	Chromosome2	Plasmid
Contig length (bp)	3,629,551	2,779,201	122,067
GC content * (%)	68.35	68.84	61.54
Total genes	3376	2262	122
Protein-coding genes	3139	2123	90
tRNAS	57	8	0
rRNAS	9	6	0
ncRNAs	4	0	0
Pseudo genes	167	125	32
GenBank accession	CP047315	CP047316	CP047317

* GC-content: the proportion of guanine-cytosine in genome.

**Table 2:** List of qPCR results from 30 proteins detected by 2D-MS/MS.

Number	Gene	Protein Name or Predicted Function/Bacterial Species	Fold Change *
A16	BCAL3235	putative UDP-galactopyranose mutase	36.76
A52	pmlR/bspR1	LuxR family transcriptional regulator	78.79
A118	SbnA	2,3-diaminopropionate biosynthesis protein	2048.00
B01	SbnB	2,3-diaminopropionate biosynthesis protein	39.40
B05	PqqE	pyrroloquinoline quinone biosynthesis protein	2521.38
B19	PqqD	pyrroloquinoline quinone biosynthesis peptide chaperone	256.00
B20	PqqC	pyrroloquinoline-quinone synthase	1024.00
B21	PqqB	pyrroloquinoline quinone biosynthesis protein	3104.19
C38	Y5A_023325	(2Fe-2S)-binding protein	157.59
E09	Y5A_015930	LysR family transcriptional regulator	238.86
E21	Y5A_026480	hypothetical protein	5404.70
E28	Y5A_005305	aldo/keto reductase	8.57
E31	Y5A_024170	benzaldehyde dehydrogenase	168.90
E46	Y5A_018250	class I SAM-dependent DNA methyltransferase	776.05
F07	flmH	3-oxoacyl-ACP reductase	128.00
F10	hemB	Porphobilinogen synthase	2048.00
F23	sodB	superoxide dismutase	84.45
G30	alcE	putative iron-sulfur protein	1024.00
G95	gmd	GDP-mannose 4,6-dehydratase	4389.98
G122	rmlC	dTDP-4-dehydrorhamnose 3,5-epimerase	10,085.54
H11	katG	catalase/peroxidase HPI	25.99
H63	algU	RNA polymerase sigma-H factor	207.94
A139	Y5A_006000	Lysine decarboxylase	2.14
B08	msrB	methionine-R-sulfoxide reductase	1.52
B27	Y5A_011315	hypothetical protein	1.41
D06	PhnA	Alkylphosphonate utilization operon protein	1.52
E13	Y5A_006405	NADH dehydrogenase subunit C	0.62
E26	Y5A_011635	aldo/keto reductase family oxidoreductase	0.66
F01	Y5A_002530	ferritin, Dps family protein	1.52
G32	Y5A_007875	Ankyrin repeat-containing protein	1.32

* The fold-change data are the expression level under human mimic medium compared to that under planta mimic medium.

**Table 3:** Strains and plasmids used in this study.

Strain or Plasmid	Relevant Characteristics ^a^	Source or Reference
***Burkholderia Glumae***		
AU6208	Human pathogen isolated from surgical specimens from lung lesions in an 8-month-old boy	Weinberg et al., 2007
LMG 2196^T^	Isolated from *Oryza sativa*, grain	Shannon Johnson, 1967
AU6208^ΔBCAL3235^	AU6208 deletion mutation defective in BCAL3235	This study
AU6208^ΔSbnAB^	AU6208 deletion mutation defective in SbnAB	This study
AU6208^ΔpmlR^	AU6208 deletion mutation defective in pmlR	This study
***Escherichia coli***		
DH5α	F^-^ Φ80d *lacZ*ΔM15Δ(*lacZYA*-*argF*) U169 *recA1 endA1, hsdR17*(r_k_^-^, m_k_^+^) *phoA supE44λ^-^ thi-1 gyrA96 relA1*	This study
S17-1(λ pir)	λ Lysogenic S17-1 derivative producing π protein for replication of plasmids carrying *oriR6K*; *recAprohsdR*RP4-2-Tc::Mu-Km::Tn7 λ^-^ pir	Simon et al., 1983
BL21(DE3)	F^-^ *ompT hsdS20*(r_b_^-^, m_b_^-^) *gal*	Novagen
**Plasmids**		
pGEM-T	Amp^R^; cloning vector	Promega
pUFR034	IncW Nm^r^ Mob^+^*mob* (P) lacZ alpha Par^+^*cos*	DeFeyter et al., 1990
pKMS1	Km^R^; R6K-based suicide vector; requires the *pir*-encoded π protein for replication	Zou et al., 2011
AU6208- pUFR034	Empty vector control	This study
pKMS1- BCAL3235	Km^R^; used to create knockout mutant of AU6208^ΔBCAL3235^	This study
pUFR034- BCAL3235	Km^R^; used to create complement of AU6208^ΔBCAL3235^	This study
pKMS1-SbnAB	Km^R^; used to create knockout mutant of AU6208^ΔSbnAB^	This study
pUFR034- SbnAB	Km^R^; used to create complement of AU6208^ΔSbnAB^	This study
pKMS1-pmlR	Km^R^; used to create knockout mutant of AU6208^ΔpmlR^	This study
pUFR034- pmlR	Km^R^; used to create complement of AU6208^ΔpmlR^	This study

^a^ Km^R^ and Amp^R^ indicate kanamycin and ampicillin resistant.

## Data Availability

All data supporting the conclusions of this article are included in this article and its additional files. The complete genome sequences have been deposited at DDBJ/EMBL/GenBank and the bioproject ID is PRJNA160245.

## Supplementary Materials

The following are available online at https://www.mdpi.com/2076-0817/10/2/87/s1, Figure S1: 2-DE maps of total proteins from *B. glumae* strain AU6208 expressed under *human mimic* and *in planta mimic* conditions. The labeled spots are the typical results of specific expressed proteins., Table S1: Unique genes in AU6208 compared to LMG 2196^T^, Table S2: Antibiotic Resistance genes in AU6208 and LMG 2196, Table S3: Virulence genes in AU6208 and LMG 2196, Table S4: Horizontal gene transfer in AU6208, Table S5: List of qPCR results from 30 proteins detected by 2D-MS/MS.
