# Supplementary figures and images for: Genome Sequence and Adaptation Analysis of the Human and Rice Pathogenic Strain Burkholderia glumae AU6208
Source: Pathogens. 2021 Jan 20;10(2):87. doi: 10.3390/pathogens10020087 (PMC7909282; doi:10.3390/pathogens10020087)

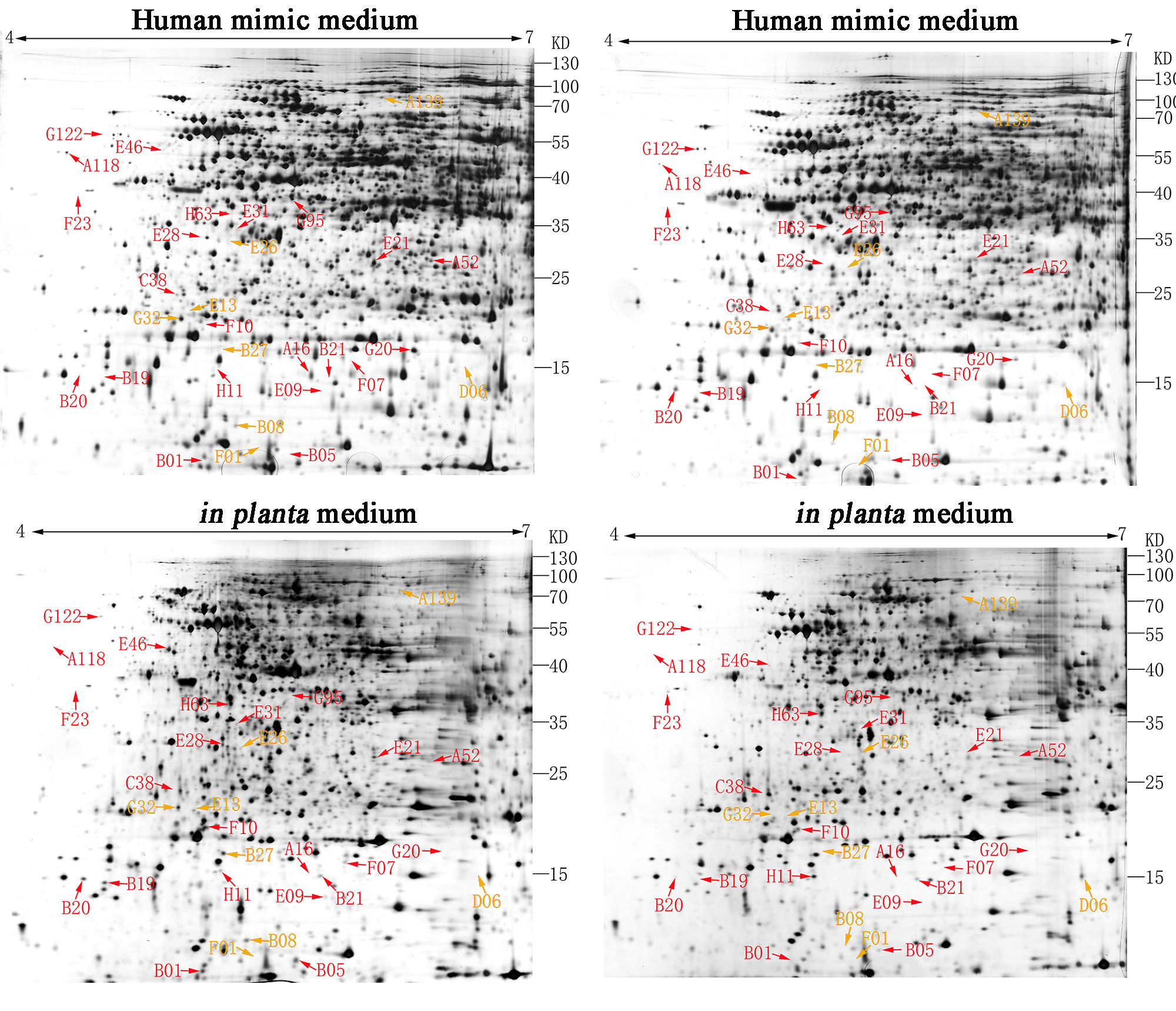

Supplement: Supplementary file 1 [file pathogens-10-00087-s001.zip › Fig. S1.jpg]
